# Long non-coding RNA titin-antisense RNA1 contributes to growth and metastasis of cholangiocarcinoma by suppressing microRNA-513a-5p to upregulate stratifin

**DOI:** 10.1080/21655979.2021.2011014

**Published:** 2021-12-13

**Authors:** Yang Liu, Jiangyang Sun, Peng Qi, Yang Liu

**Affiliations:** a Department of Hepatobiliary Surgery, Huanggang Center Hospital, Huanggang, Hubei, China; b Department of Hepatobiliary Surgery, The Central Hospital of Wuhan, Tongji Medical College, Huazhong University of Science and Technology, Wuhan, Hubei, China; c Department of General Surgery, Hubei No. 3 People’s Hospital of Jianghan University, Wuhan, Hubei, China

**Keywords:** Cholangiocarcinoma, lncRNA, TTN-AS1, miR-513a-5p, SFN

## Abstract

Cholangiocarcinoma (CCA) is one of the most common histological types of primary hepatic malignancy and is associated with poor overall prognosis, causing a ponderous burden on human life. Hence, it is necessary to elucidate the pathogenesis of CCA. The objective of our research was to shed light on the mechanism through which long non-coding RNA titin-antisense RNA1 (lncRNA TTN-AS1) is involved in the development of CCA. Reverse transcription quantitative polymerase chain reaction was used to detect TTN-AS1 expression in CCA samples and cells. Functional experiments were performed using the Cell Counting Kit-8, 5-ethynyl-2ʹ-deoxyuridine, transwell, and *in vivo* tumor growth assays. The relationship between TTN-AS1, miR-513a-5p, and stratifin (SFN) was explored using a dual luciferase reporter assay, RNA immunoprecipitation (RIP) experiment, and Pearson correlation analysis. The result showed that TTN-AS1 and SFN are highly expressed in CCA tissues. Bioinformatics analysis, luciferase reporter and RIP experiments revealed the correlation between TTN-AS1, miR-513a-5p, and SFN. In addition, silencing TTN-AS1 mitigated CCA cell proliferation and migration. Mechanistically, miR-513a-5p is sponged by TTN-AS1. The miR-513a-5p inhibitor abolished the effect of TTN-AS1 silencing on the aggressive behaviors of CCA cells. Furthermore, we showed that miR-513a-5p is a regulator of CCA by targeting SFN. TTN-AS1 induced CCA cell growth and metastasis via the miR-513a-5p/SFN pathway, which offers a new strategy for therapeutic interventions for CCA.

## Introduction

Cholangiocarcinoma (CCA) is an aggressive malignant tumor derived from cholangiocytes, and ranks second among the most common primary hepatic carcinomas [1]. Although surgical excision is a relatively effective strategy for the treatment of early CCA, the vast majority of patients are initially diagnosed with advanced CCA owing to the lack of distinctive clinical features and timely diagnosis in the early stages [2,3]. Even worse, the recurrence rate of patients undergoing radical surgery reaches 65%–75% [4]. Therefore, gaining a deep understanding of the underlying pathogenesis of gastric cancer is indispensable for the improvement of CCA therapy.

Long non-coding RNAs (lncRNAs) are RNA molecules containing over 200 nucleotides that lack protein-coding ability [5]. Over the past few years, the roles of lncRNAs in tumor progression has attracted the attention of researchers [6]. LncRNAs affect cell biological processes via chromosomal modification, genetic silencing, transcriptional regulation, and nuclear transport [7,8]. Dysregulation of lncRNAs has been shown to be closely associated with the occurrence and development of numerous malignancies, including CCA [9–11]. Accumulating reports reveal that a variety of lncRNAs are tumorigenic or cancer suppressor genes in CCA [12,13]. Titin-antisense RNA1 (TTN-AS1), located on chromosome 2q12.2, arises from the opposite strand of TTN [14]. Many studies have demonstrated that abnormally expressed TTN-AS1 is involved in the progression of multiple cancers [15]. For instance, lncRNA TTN-AS1 induces the development and metastasis of cutaneous skin melanoma by stabilizing TTN mRNA [16]. TTN-AS1 acts as a promoting factor in ovarian cancer via the miR-139-5p/ROCK2 axis [17]. TTN-AS1 upregulates Dickkopf Wnt signaling pathway inhibitor 1 (DKK1) to facilitate osteosarcoma progression by competitively binding to microRNA-376a [18]. Moreover, recent reports suggest that TTN-AS1 is upregulated in CCA, and promotes proliferation, migration, and *in vivo* growth of CCA cells [19]. However, the regulatory network of TTN-AS1 in CCA needs to be further elucidated.

This study aimed to elucidate the mechanism through which TTN-AS1 is involved in the development of CCA. We hypothesized that TTN-AS1 promotes cell proliferation and migration during CCA progression by modulating the miR-513a-5p/SFN axis, which may be a new therapeutic target for patients with CCA.

## Materials and methods

### Sample collection and cell culture

CCA tissues and adjacent normal samples were obtained from 30 patients hospitalized in the Huanggang Center Hospital (Huanggang, Hubei, China). None of the participants received any treatment prior to the surgery. All enrolled patients signed written informed consent forms. The procedures in this study were in line with the Helsinki Declaration, and this study was approved by the ethics committee of Huanggang Center Hospital. Patient characteristics are listed in Table 1.

**Table 1.** Clinical characteristics of 30 cholangiocarcinoma patients

Parameters	N (30, %)
Age (years)	
≥61	18 (60.00%)
<61	12 (40.00%)
Gender	
Male	24 (80.00%)
Female	6 (20.00%)
Tumor stage	
I–II	21 (70.00%)
III–IV	9 (30.00%)
Differentiation	
High	2 (6.67%)
Moderate	16 (53.33%)
Low	12 (40.00%)
Location	
Intrahepatic	27 (90.00%)
Extrahepatic	3 (10.00%)
CA19-9	
≥100 U/mL	9 (30.00%)
<100 U/mL	21 (70.00%)
AFP	
Positive (≥20 ng/mL)	25 (83.33%)
Negative (<20 ng /mL)	5 (16.67%)

Tumor stage was defined according to the TNM classification for CCA in the American Joint Committee on Cancer in the cancer staging manual (8th edition); CA19-9, carbohydrate antigen 19–9; AFP, alpha‐fetoprotein.

The human biliary epithelial cell line HIBEC and four human CCA cell lines (TFK-1, CCLP, HCCC-9810, and HUCCT1) were acquired from the Shanghai Institute of Cell Biology (Shanghai, China) and cultivated in RPMI-1640 medium (HyClone, USA) containing 10% fetal bovine serum (HyClone) and 1% penicillin/streptomycin (Gibco, USA) in a humidified incubator containing 5% CO_2_ at 37°C.

### Reverse transcription-quantitative PCR (RT-qPCR)

After RNA extraction from clinical specimens and cells using TRIzol (Invitrogen, USA), cDNA synthesis was performed using the PrimeScript™ RT Master Mix Kit (Takara, Japan). RT-qPCR was carried out on a PCR Detection Apparatus (Agilent Technologies, USA) using SYBR Green qPCR SuperMix (Takara). The 2^−ΔΔCt^ method was used to calculate the gene expression [20]. The expression of miRNAs was normalized to Uracil6 (U6), and glyceraldehyde-3-phosphate dehydrogenase (GAPDH) served as an endogenous control for lncRNA TTN-AS1 and mRNAs. The primers used are shown in Table 2.

**Table 2.** The primers used in the presence work

Names	Primers	Sequences (5ʹ to 3ʹ)
TTN-AS1	Forward	GCCAGGTAGAGTTGCAGGTT
	Reverse	GAAGCTGCTGCGGATGAATG
miR-513a-5p	Forward	TAAATTTCACCTTTCTGAGAAGG
	Reverse	GCGAGCACAGAATTAATACGAC
miR-494-3p	Forward	ATCCAGTGCGTGTCGTG
	Reverse	GCGAGCACAGAATTAATACGAC
miR-2278	Forward	GAGAGCAGTGTGTGTTGCCT
	Reverse	GAACATGTCTGCGTATCTC
miR-2114-5p	Forward	TAGTCCCTTCCTTGAAGCGG
	Reverse	GAACATGTCTGCGTATCTC
SFN	Forward	TCCACTACGAGATCGCCAACAG
	Reverse	GTGTCAGGTTGTCTCGCAGCA
CDKN2A	Forward	CAACGCACCGAATAGTTACG
	Reverse	CAGCTCCTCAGCCAGGTC
GAPDH	Forward	GAGTCCACTGGCGTCTTC
	Reverse	GTTGAGGTCAATGAAGGG
U6	Forward	CTCGCTTCGGCAGCACA
	Reverse	AACGCTTCACGAATTTGCGT

### Subcellular localization analysis

The cytoplasmic/nuclear RNA separation and purification Kit (Sigma, USA) was used to isolate and purify cytoplasmic and nuclear fractions, according to the manufacturer’s instructions. The lncRNA TTN-AS1 was detected by RT-qPCR assay, with GAPDH as the cytoplasmic endogenous reference and U6 as the nuclear endogenous reference [21].

### Cell transfection

For knockdown of TTN-AS1 or SFN, specific short hairpin RNAs (sh-RNAs) against TTN-AS1 (sh-TTN-AS1) or SFN (sh-SFN) were produced by Genepharma (Shanghai, China) and nonspecific sh-RNAs were used as the negative controls. The oligonucleotides of miR-513a-5p mimic and inhibitor, as well as the corresponding negative control NC mimic and inhibitor, were also acquired from Genepharma. The indicated plasmids and oligonucleotides were transfected into cells using Lipofectamine 2000 (Invitrogen) according to the manufacturer’s instructions.

### Cell proliferation assay

The Cell Counting Kit-8 (CCK-8; Beyotime, Shanghai, China) assay was used to determine cell viability. After transfection, CCA cells were seeded into 96-well plates at a density of 2 × 10^3^ cells/well. At 24, 48, 72, and 96 h post incubation at 37°C, CCA cells were treated with CCK-8 reagent and incubated for another 2 h. The absorbance was measured at 450 nm using a microplate reader (BioTek Instruments, USA) [22].

### 5-ethynyl-2ʹ-deoxyuridine (EdU) staining assay

The Cell-Light™ EdU Cell Proliferation Detection Assay (Life, USA) was used to assess proliferation. Briefly, CCLP and HCCC-9810 cells were incubated with 50 nM EdU for 2 h. Subsequently, the cells were fixed with 4% formaldehyde at room temperature for 1 h and treated with 0.5% Triton x-100 for 15 min for permeabilization. After washing with PBS, the cells were reacted with Apollo reaction cocktail for 30 min and stained with 4ʹ, 6-diamidino-2-phenylindole (DAPI) for 30 min. Finally, cells were observed under a fluorescence microscope, and the percentage of EDU-positive cells was counted.

### Transwell migration assay

The migration of CCLP and HCCC-9810 cells was assayed by the transwell assay using transwell chambers with 8 μm pores (Corning Inc., USA). Transfected CCLP and HCCC-9810 cells were resuspended in 100 μl serum-free RPMI-1640 medium and placed in the upper chamber of the transwell. Subsequently, the bottom chambers were filled with 500 μl RPMI-1640 medium containing 20% FBS. Following incubation for 24 h, cells passing through the membrane were immobilized with 4% paraformaldehyde, stained with 0.1% crystal violet, and visualized by microscopy [23].

### RNA immunoprecipitation (RIP) assay

The EZMagna RIP kit (Millipore, USA) was used to conduct the RIP experiments based on the manufacturer’s protocol. Briefly, CCLP and HCCC-9810 cells were lysed using RIP lysis buffer, and immunoprecipitation was performed using anti-Ago2 antibody and magnetic beads, and anti-IgG was used as a negative control. Thereafter, RNA was eluted from the magnetic beads, purified, and analyzed by RT-qPCR [24].

### Xenograft experiments

Animal experiments were conducted with the approval of the Animal Research Ethics Committee of the Huanggang Center Hospital. After one week of adaptive feeding in a pathogen-free environment with 50% humidity at 28°C, BALB/c nude mice were subcutaneously inoculated with 5 × 10^6^ CCLP cells stably transfected with sh-TTN-AS1 or sh-NC. After 5 weeks, the mice were euthanized, and neoplasms were dissected from nude mice and weighed. The tumor size was monitored and measured every week [25].

### Dual-luciferase reporter assay

For pmirGLO-TTN-AS1-wild-type (wt) and pmirGLO-TTN-AS1-mutant (mut) constructs, the sequences of wild-type and mutant TTN-AS1 were subcloned into dual-luciferase reporter plasmids. Furthermore, pmirGLO-SFN-wt and pmirGLO-SFN-mut vectors were obtained in the same manner. CCLP and HCCC-9810 cells were co-transfected with the reporter vectors and miR-513a-5p mimic or negative control NC mimic using Lipofectamine 2000 (Invitrogen). Finally, luciferase activity was determined using the Dual-Luciferase Reporter Detection System (Promega, USA) in conformity with the supplier’s directions at 48 h after transfection [23].

### Western blot

Total protein from CCA tissue samples and cells was isolated using radioimmunoprecipitation assay (RIPA) lysis buffer (Beyotime) and quantified using a bicinchoninic acid (BCA) protein detection kit (Beyotime) according to the manufacturer’s instructions. Protein samples were electrophoretically separated by 10% sulfate-polyacrylamide gel electrophoresis (SDS-PAGE) and transferred onto polyvinylidene fluoride (PVDF) membranes (Millipore). Membranes were then incubated in 5% defatted milk, probed with primary antibodies against SFN and GAPDH (Abcam, USA) overnight, and then incubated with appropriate secondary antibodies. GAPDH was used as an internal control. Protein bands were visualized with an enhanced chemiluminescence kit (Beyotime), following the manufacturer’s instructions [26].

## Statistical analysis

Data processing and analysis were performed using SPSS software (version 20.0; IBM, USA). Experimental results are represented as the mean ± standard deviation from three independent assays. Comparisons between two groups were performed using Student’s t-test. One-way analysis of variance (ANOVA) was used to assess differences among three or more groups. The correlation between genes was estimated using the Pearson correlation analysis. Differences were considered statistically significant at *P* < 0.05.

## Results

Here, we aimed to explore the role of TTN-AS1 in CCA. We conducted a series of *in vitro* and *in vivo* experiments and found that TTN-AS1 promoted the proliferation, migration, and *in vivo* growth of CCA cells by targeting miR-513a-5p and releasing SFN. Therefore, the function of the TTN-AS1/miR-513a-5p and/SFN axis in CCA was studied for the first time, which provides a new insight into the pathogenesis of CCA.

### TTN-AS1 might regulate SFN in CCA by sponging miR-513a-5p

**Figure 1.**
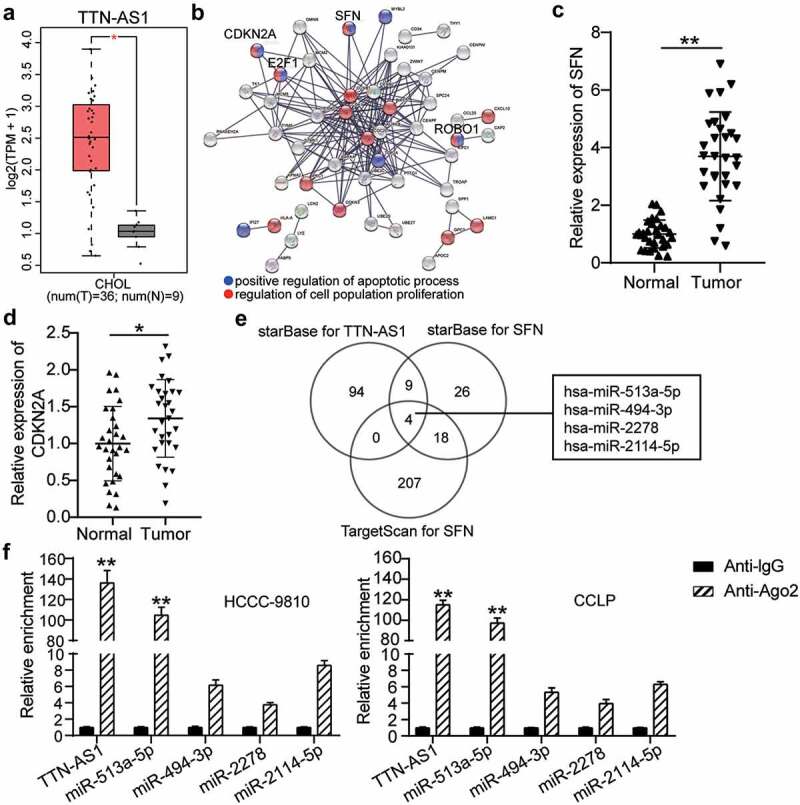
**The identification of TTN-AS1/miR-513a-5p/SFN axis in cholangiocarcinoma**. (a) The expression of TTN-AS1 in cholangiocarcinoma based on GEPIA data. T, tumor. N. normal. (b) The upregulated genes from GEPIA were performed the GO enrichment by STRING. (c-d) The expression of SFN and CDKN2A in tumor tissues (N = 30) and normal tissues (N = 30). **P < 0.01 vs. normal control group. (e) The miRNAs targeting TTN-AS1 and SFN were predicted by starBase and TargetScan. (f) The enrichment of TTN-AS1 in four miRNAs was detected by RIP assay. **P < 0.01 vs. anti-IgG group

Analysis of the GEPIA database indicated that TTN-AS1 was upregulated in CCA specimens (Figure 1a) and has been reported to promote CCA progression [27]. Therefore, TTN-AS1 was selected as a key lncRNA for further exploration. After GO enrichment of upregulated genes in CCA from the GEPIA database, the STRING results showed that SFN, cyclin dependent kinase inhibitor 2A (CDKN2A), E2F transcription factor 1 (E2F1), and roundabout guidance receptor 1 (ROBO1) were related to cell apoptosis and proliferation (Figure 1b). Because previous studies have explored the effects of E2F1 and ROBO1 on CCA [28,29], SFN and CDKN2A attracted our attention. SFN and CDKN2A expression was found to be significantly upregulated in CCA tissues (Figure 1c and d). Then, starBase was employed to predict the binding of miRNAs with TTN-AS1 (Gene ID: ENSG00000237298) and SFN, and TargetScan was used to identify the miRNAs with affinity to SFN. As shown in Figure 1e, the four miRNAs overlapped (Figure 1e). To further identify the key miRNAs, an RIP assay was performed, which revealed that TTN-AS1 was mainly enriched by miR-513a-5p (figure 1f).

### Silencing TTN-AS1 inhibits CCA cell proliferation and migration

**Figure 2.**
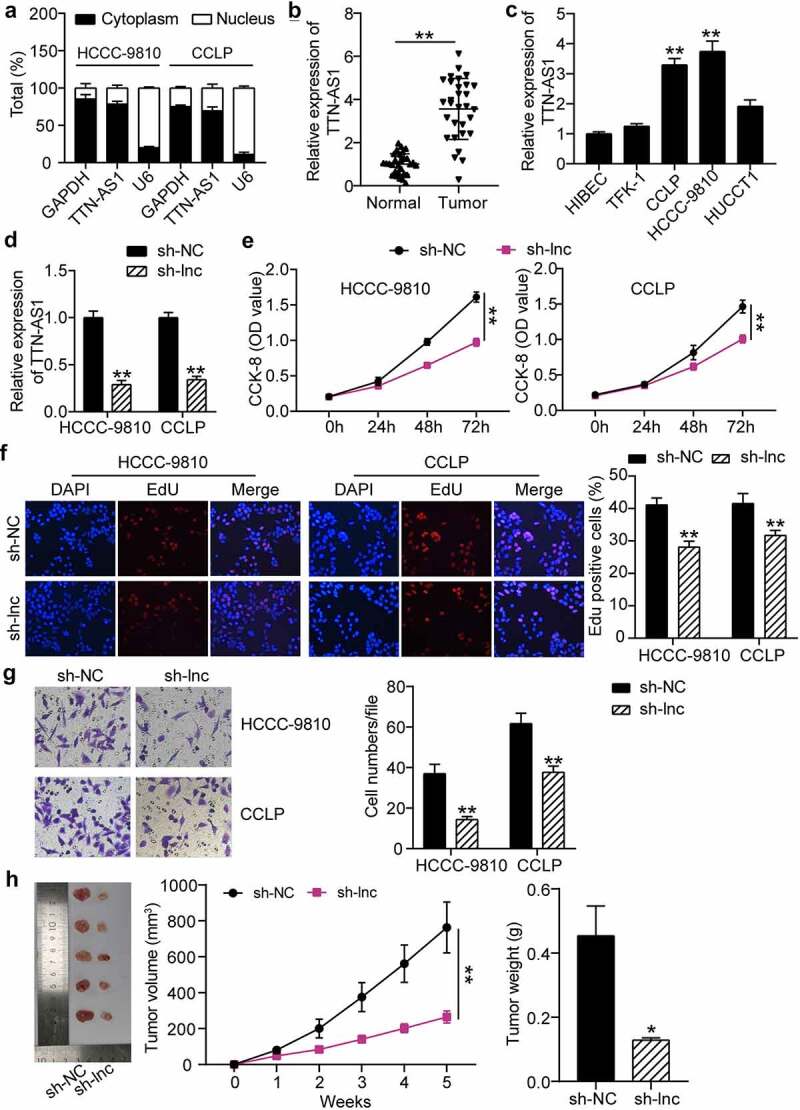
**Silencing of TTN-AS1 inhibited the proliferation and migration of cholangiocarcinoma cells**. (a) The subcellular localization of TTN-AS1 in CCLP and HCCC-9810 cells. (b) The RT-qPCR detection of TTN-AS1 level in tumor tissues (N = 30) and normal samples (N = 30) from cholangiocarcinoma patients. **P < 0.01 vs. normal control group. (c) The expression of TTN-AS1 in cholangiocarcinoma cells (TFK-1, CCLP, HCCC-9810 and HUCCT1) and biliary epithelia HIBEC. **P < 0.01 vs. HIBEC. (d) The transfection efficiency of sh-TTN-AS1 was measured by RT-qPCR. **P < 0.01 vs. sh-NC group. (e) The proliferation of CCLP and HCCC-9810 cells was detected by CCK-8 assay. vs. sh-NC group, **P < 0.01. (f) The EdU percentage of CCLP and HCCC-9810 cells was detected by EdU assay. **P < 0.01 vs. sh-NC group. (g) Transwell assay was conducted to estimate cell migration. (h) The volume and weight of tumors were detected after 5 weeks of xenograft. *P < 0.05, **P < 0.01 vs. sh-NC group

To validate our hypothesis, a subcellular fractionation analysis was conducted to determine the localization of TTN-AS1. As expected, TTN-AS1 was preferentially expressed in the cytoplasm of CCA cells (Figure 2a). Results of the RT-qPCR assay revealed that TTN-AS1 levels were higher in CCA samples than in matched normal specimens (Figure 2b). Likewise, TTN-AS1 expression was overtly augmented in CCA cells, in contrast to normal biliary epithelial cells (Figure 2c). Considering that CCLP and HCC-9810 cells presented the highest TTN-AS1 expression, these two cell lines were selected for subsequent experiments. Subsequently, TTN-AS1 was silenced in the indicated cells for loss-of-function assays, and the effectiveness of TTN-AS1 knockdown was confirmed by RT-qPCR (Figure 2d). The CCK-8 assay revealed that TTN-AS1 depletion significantly reduced the proliferation of CCLP and HCC-9810 cells (Figure 2e). The EdU assay revealed that TTN-AS1 knockdown inhibited the percentage of EdU positive cancer cells (Figure 2f). The Transwell assay revealed that downregulation of TTN-AS1 repressed CCA cell migration (Figure 2g). Consistently, silencing TTN-AS1 led to a significant decrease in the size and weight of xenograft tumors, confirming that silencing of TTN-AS1 restrained cell growth *in vivo* (Figure 2h). Collectively, our results indicated that TTN-AS1 facilitated CCA cell growth and metastasis.

### TTN-AS1 is a miRNA sponge for miR-513a-5p

**Figure 3.**
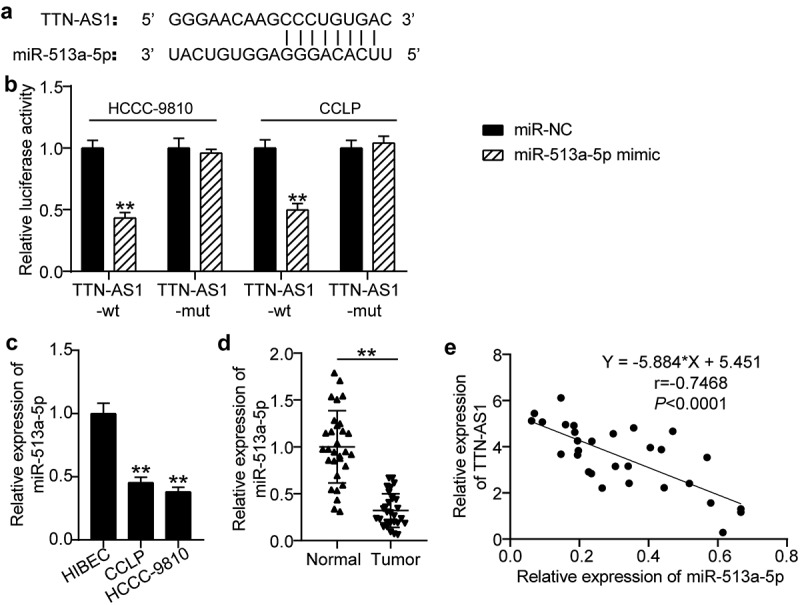
**TTN-AS1 was a miRNA sponge for miR-513a-5p**. (a) The potential binding sites between TTN-AS1 and miR-513a-5p. (b) Dual-luciferase reporter assay was adopted to confirm the predicted relationship. **P < 0.01 vs. miR-NC group. (c) The expression of miR-513a-5p in normal cells and cholangiocarcinoma cells. **P < 0.01 vs. HIBEC. (d) The RT-qPCR analysis of miR-513a-5p level in clinical specimens. **P < 0.01 vs. normal control group. (e) Pearson correlation analysis of TTN-AS1 and miR-513a-5p

As shown in Figure 3a, miR-513a-5p harbored the predicted binding sites for TTN-AS1. Considering the co-existence of TTN-AS1 and miR-513a-5p in RNA-induced silencing complex (RISC) was demonstrated by the RIP experiment, a dual-luciferase reporter assay was used for further verification. Our observations revealed that the luciferase activity of TTN-AS1-wt was only weakened by the miR-513a-5p mimic, while there was no remarkable change in the luciferase activity of the mutant forms (Figure 3b). Additionally, miR-513a-5p was downregulated in CCLP and HCC-9810 cells compared to that in normal cells (Figure 3c). In contrast to normal tissues from CCA patients, miR-513a-5p expression was decreased in tumor samples (Figure 3d). Pearson correlation analysis further showed the negative association between miR-513a-5p and TTN-AS1 (Figure 3e). Thus, miR-513a-5p is a target of TTN-AS1.

### Suppression of miR-513a-5p abolishes the effect of TTN-AS1 knockdown in CCA cell proliferation and migration

**Figure 4.**
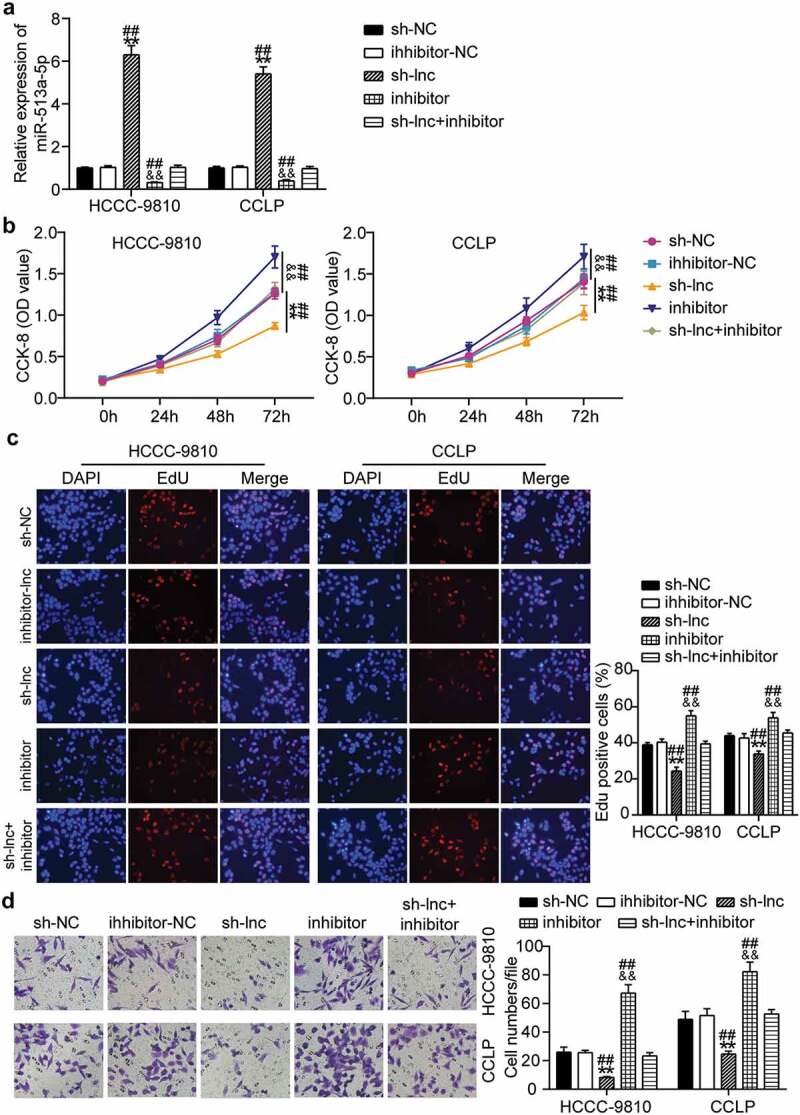
**Suppression of miR-513a-5p abolished the role of TTN-AS1 knockdown in cholangiocarcinoma cell proliferation and migration**. (a) The effects of TTN-AS1 on miR-513a-5p expression was evaluated by RT-qPCR. (b) CCK-8 assay was performed to determine the role of miR-513a-5p in cell proliferation. (c) The EdU percentage of CCLP and HCCC-9810 cells regulated miR-513a-5p was detected by EdU assay. (d) The potential of miR-513a-5p in cell migratory capability was assessed by transwell assay. **P < 0.01 vs. sh-NC group. ^&&^*P* < 0.01 vs. inhibitor-NC. ^##^*P* < 0.01 vs. sh-lnc+inhibitor

Subsequently, we determined whether TTN-AS1 participates in CCA progression via miR-513a-5p. RT-qPCR results showed that TTN-AS1 inhibition markedly enhanced miR-513a-5p expression, and suppression of miR-513a-5p abolished the effects of TTN-AS1 silencing on miR-513a-5p expression (Figure 4a). According to the CCK-8 assay, miR-513a-5p inhibitor promoted CCA cell proliferation and recovered the proliferative ability of CCLP and HCC-9810 cells repressed by depletion of TTN-AS1 (Figure 4b). EdU analysis revealed that miR-513a-5p inhibitor increased the rate of EdU-positive CCLP and HCC-9810 cells, and reversed the reduction in EdU-positive rate caused by TTN-AS1 knockdown (Figure 4c). Similarly, miR-513a-5p inhibitor promoted the migration of CCLP and HCC-9810 cells, and the inhibitory effects of TTN-AS1 downregulation on cell migration were abrogated by miR-513a-5p inhibitor (Figure 4d). Based on the above findings, we concluded that TTN-AS1 serves as an oncogene in CCA via miR-513a-5p.

### SFN acts as a downstream effector of miR-513a-5p

**Figure 5.**
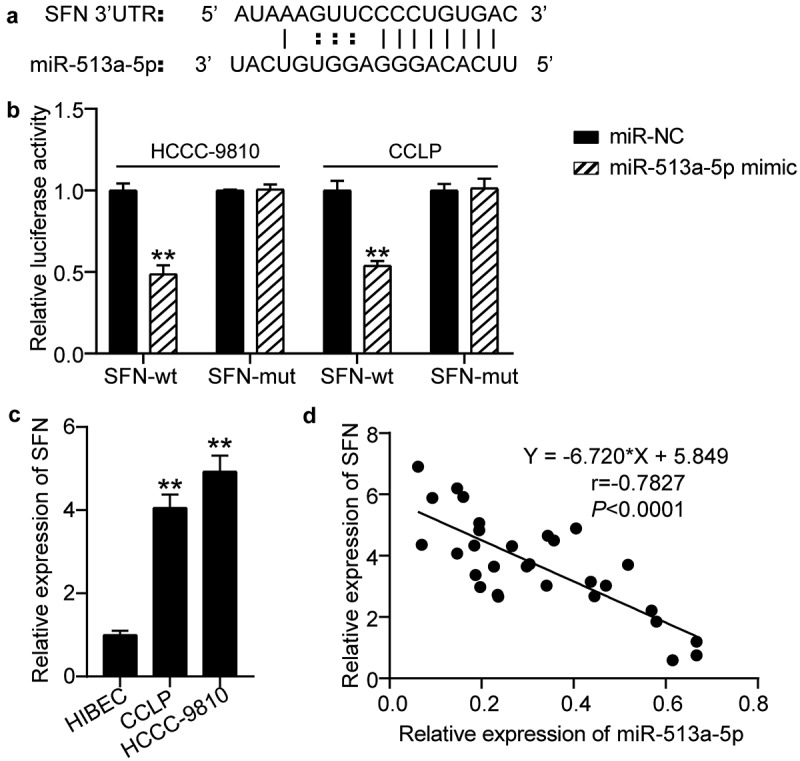
**SFN acted as a downstream effector of miR-513a-5p**. (a) The predicted miR-513a-5p binding sites in 3ʹUTR of SFN. (b) The interplay of miR-513a-5p and SFN was validated by dual-luciferase reporter assay. vs. miR-NC group, **P < 0.01. (c) The expression of SFN in normal cells HIBEC as well as cholangiocarcinoma cells CCLP and HCCC-9810. vs. HIBEC, **P < 0.01. (d) Pearson correlation analysis of SFN and miR-513a-5p

By browsing the starBase website, we uncovered potential binding sites for miR-513a-5p in the 3ʹUTR of SFN (Figure 5a). Accordingly, we performed a dual-luciferase reporter assay to confirm their relationship. Our findings showed that co-transfection of miR-513a-5p mimic and wild-type SFN resulted in a conspicuous decrease in luciferase activity in CCLP and HCC-9810 cells, confirming that miR-513a-5p directly bound to SFN (Figure 5b). RT-qPCR data indicated that CCLP and HCC-9810 cells exhibited higher SFN expression levels than HIBEC cells (Figure 5c). Pearson correlation analysis suggested that miR-513a-5p was negatively correlated with SFN (Figure 5d). Thus, SFN is targeted by miR-513a-5p.

### SFN mediates the function of miR-513a-5p in the malignant behaviors of CCA cells

**Figure 6.**
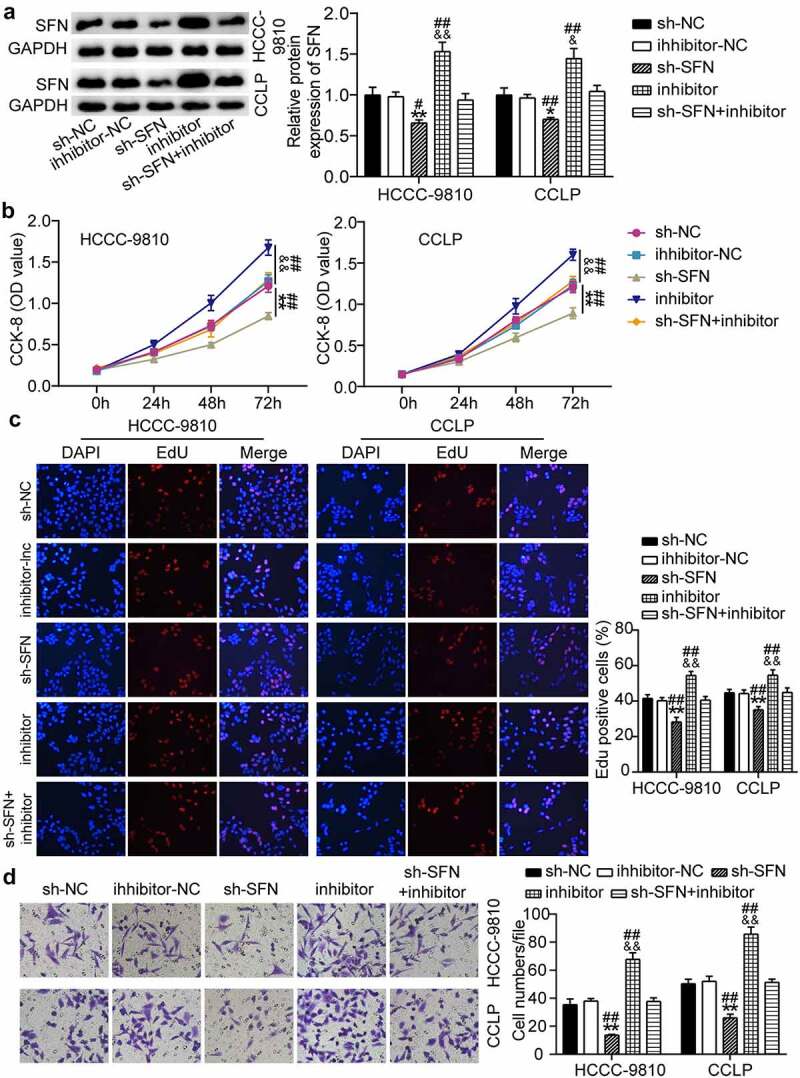
**SFN was responsible for the function of miR-513a-5p in the malignant behaviors of cholangiocarcinoma cells**. (a) RT-qPCR analysis was employed to detect the impacts of miR-513a-5p on SFN expression. (b) The influences of SFN on cell proliferation were identified by CCK-8 assay. (c) The EdU percentage of CCLP and HCCC-9810 cells regulated SFN was detected by EdU assay. (d) Transwell assay was conducted to ascertain the role of SFN in cell migration. *P < 0.05, **P < 0.01 vs. sh-NC group. ^&&^*P* < 0.01 vs. inhibitor-NC. ^##^*P* < 0.01 vs. sh-SFN+inhibitor

Based on these findings, we investigated whether miR-513a-5p plays a role in the biological processes of CCA by targeting SFN. On the basis of Western blot results, SFN was upregulated owing to miR-513a-5p suppression, and then the recovery of SFN levels occurred when SFN was knocked down (Figure 6a). The CCK-8 assay revealed that silencing of SFN reduced the viability of CCLP and HCC-9810 cells, and miR-513a-5p inhibitor-induced cell proliferation was reversed by SFN downregulation (Figure 6b). In addition, knocking down SFN restrained the rate of EdU-positive CCLP and HCC-9810 cells and partially reduced the increase in the EdU-positive rate caused by the miR-513a-5p inhibitor (Figure 6c). In agreement with the above-mentioned data, CCA cell migration was repressed by SFN knockdown and miR-513a-5p inhibitor-induced cell migration was abrogated by the depletion of SFN (Figure 6d). These results indicate that SFN mediates the effect of miR-513a-5p on CCA.

## Discussion

In the current study, we confirmed that lncRNA TTN-AS1 is highly expressed in CCA. Loss-of-function assays revealed that silencing TTN-AS1 impedes CCA cell proliferation and migration and inhibits tumor growth *in vivo*. Mechanistically, TTN-AS1 functioned as a sponge for miR-513a-5p. Our findings showed that the repression of miR-513a-5p contributed to the malignancy of CCA cells and reversed the effect of TTN-AS1 in CCA. Furthermore, SFN served as a downstream effector of miR-513a-5p and mediated its effects on CCA cell proliferation and migration.

Many studies have shown that lncRNAs function as key players in the tumorigenicity of diverse malignant tumors, including CCA [8,11,30]. Aberrant lncRNA expression has been reported to be strongly associated with CCA progression [12,31,32]. Accumulating evidence suggests that lncRNA TTN-AS1 promotes a variety of malignancies, including lung carcinoma, breast cancer, colorectal cancer, gastric carcinoma, and other common cancers [33] [14,34–36]. For instance, TTN-AS1 contributes to cell proliferation and drug resistance in osteosarcoma by targeting the miR-134-5p/mbt domain containing 1 (MBTD1) pathway [37]. The lncRNA TTN-AS1 upregulates Kruppel-like factor 15 (KLF15) expression to accelerate colorectal cancer development by sponging miR-376a-3p [38]. TTN-AS1 promotes proliferation, migration, invasion, and epithelial-mesenchymal transformation (EMT) of hepatocellular carcinoma cells by regulating the miR-139-5p/ sparc/osteonectin, cwcv, and kazal-like domains proteoglycan 1 (SPOCK1) axis [39]. More importantly, TTN-AS1 promotes tumorigenesis and the development of CCA [19]. In agreement with these results, we confirmed that TTN-AS1 expression was prominently elevated in CCA clinical tissues and cell lines, and TTN-AS1 induced malignant behaviors of CCA cells. Likewise, silencing of TTN-AS1 retarded the growth of CCA cells *in vivo*.

It is widely documented that miR-513a-5p is a critically involved in the development of various diseases, especially tumors [40–45]. Numerous studies have shown that miR-513a-5p plays an inhibitory role in a wide range of cancers [46–48]. For example, miR-513a-5p enhances the radiosensitivity of osteosarcoma cells by suppressing apurinic/apyrimidinic endonuclease [49]. MiR-513a-5p restrains the proliferation and glycolysis of colorectal cancer cells by repressing hexokinase 2 expression [50]. Nonetheless, the effects of miR-513a-5p on CCA remain unclear. In view of the fact that TTN-AS1 predicted to harbor binding sites for miR-513a-5p, we selected miR-513a-5p for in-depth investigation. Our findings revealed that TTN-AS1 directly binds to miR-513a-5p and is negatively related to miRNA. Additionally, inhibition of miR-513a-5p retarded CCA tumorigenesis, and the effect of TTN-AS1 in CCA was mediated by miR-513a-5p.

SFN has been used as a prognostic biomarker in human cancer [51,52]. Furthermore, abnormally expressed SFN accelerates tumorigenesis and the progression of multiple malignancies [53–55]. Shiba-Ishii et al. indicated that SFN expedited lung cancer development [56]. Consistently, Hu et al. revealed that SFN is responsible for the carcinogenic role of LINC01128 in cervical cancer [57]. However, there are no reports on the involvement of SFN in CCA. Considering that SFN is a candidate target of miR-513a-5p, we further explored the interplay between SFN and miR-513a-5p. As expected, our results indicated that SFN functions as a downstream target of miR-513a-5p. SFN aggravated the aggressive features of CCA cells and was responsible for the effects of miR-513a-5p on CCA development.

This study provides strong evidence that TTN-AS1 promotes the malignancy of CCA and offers novel insights into the mechanism underlying the role of TTN-AS1 in CCA. However, there are limitations to this study. In particular, *in vivo* assays are needed to confirm the impact of TTN-AS1 on metastasis in CCA. Hence, animal experiments will be performed in a subsequent study.

## Conclusion

In conclusion, we verified that TTN-AS1 facilitates CCA progression by targeting miR-513a-5p-mediated SFN. To our knowledge, our study is the first to unravel the role of TTN-AS1/miR-513a-5p/SFN pathway in CCA pathogenesis, which might be a promising therapeutic target for patients with CCA.

## Data Availability

The datasets used and/or analyzed during the current study are available from the corresponding author on reasonable request.

## Highlight

- TTN-AS1 and SFN were highly expressed in cholangiocarcinoma, while miR-513a-5p showed low expression.
- Silencing TTN-AS1 mitigated cholangiocarcinoma cell proliferation and migration.
- miR-513a-5p was sponged by TTN-AS1.
- miR-513a-5p was a regulator of cholangiocarcinoma through targeting SFN.

## Code availability

Not available.
